# Neutrophils Isolated from Septic Patients Exhibit Elevated Uptake of Vitamin C and Normal Intracellular Concentrations despite a Low Vitamin C Milieu

**DOI:** 10.3390/antiox10101607

**Published:** 2021-10-13

**Authors:** Anitra C. Carr, Stephanie Bozonet, Juliet Pullar, Emma Spencer, Patrice Rosengrave, Geoff Shaw

**Affiliations:** 1 Department of Pathology and Biomedical Science, University of Otago, Christchurch 8140, New Zealand; stephanie.bozonet@otago.ac.nz (S.B.); juliet.pullar@otago.ac.nz (J.P.); emma.spencer@otago.ac.nz (E.S.); patrice.rosengrave@otago.ac.nz (P.R.); 2 Centre for Postgraduate Nursing Studies, University of Otago, Christchurch 8140, New Zealand; 3 Department of Intensive Care Medicine, Christchurch Hospital, Private Bag 4710, Christchurch 8140, New Zealand; geoff.shaw@cdhb.health.nz

**Keywords:** ascorbate, vitamin C, dehydroascorbic acid, sepsis, septic shock, neutrophils, erythrocytes, myeloperoxidase

## Abstract

Vitamin C (ascorbate) plays an important role in neutrophil function and is accumulated by the cells either directly via vitamin C transporters (SVCT) or indirectly following oxidation to dehydroascorbic acid. Septic patients are known to have significantly depleted plasma ascorbate status, but little is known about the ascorbate content of their circulating cells. Therefore, we assessed the ascorbate concentrations of plasma, leukocytes and erythrocytes from septic patients and compared these to healthy controls. Non-fasting blood samples were collected from healthy volunteers (*n* = 20) and critically ill patients with sepsis (*n* = 18). The ascorbate content of the plasma and isolated neutrophils and erythrocytes was measured using HPLC and plasma myeloperoxidase concentrations were determined using ELISA. Ex vivo uptake of ascorbate and dehydroascorbic acid by neutrophils from septic patients was also assessed. Neutrophils isolated from septic patients had comparable intracellular ascorbate content to healthy volunteers (0.33 vs. 0.35 nmol/10^6^ cells, *p* > 0.05), despite significantly lower plasma concentrations than the healthy controls (14 vs. 88 µmol/L, *p* < 0.001). In contrast, erythrocytes from septic patients had significantly lower intracellular ascorbate content than healthy controls (30 vs. 69 µmol/L, *p* = 0.002), although this was 2.2-fold higher than the matched plasma concentrations in the patients (*p* = 0.008). Higher concentrations of myeloperoxidase, a source of reactive oxygen species, were observed in the septic patients relative to healthy controls (194 vs. 14 mg/mL, *p* < 0.0001). In contrast to neutrophils from healthy volunteers, the neutrophils from septic patients demonstrated elevated uptake of extracellular ascorbate. Overall, neutrophils from septic patients exhibited comparable intracellular ascorbate content to those from healthy controls, despite the patients presenting with hypovitaminosis C. The mechanisms involved are currently uncertain, but could include increased generation of dehydroascorbic acid in septic patients, enhanced basal activation of their neutrophils or upregulation of their vitamin C transporters.

## 1. Introduction

Neutrophils are one of the body’s primary innate immune defences against invading pathogens. During infections, circulating neutrophils increase in number and the cells migrate from the bloodstream into infected tissues where they proceed to phagocytose and kill the invading pathogens [1]. Severe infections can progress to sepsis, life-threatening organ dysfunction due to a dysregulated host response to infection, and septic shock, characterised by profound circulatory, cellular, and metabolic abnormalities and high mortality rates [2]. Furthermore, patients with sepsis and septic shock are known to be under enhanced systemic oxidative stress, as determined by elevated concentrations of circulating oxidative stress biomarkers and depleted concentrations of important antioxidants such as vitamin C [3,4].

Vitamin C (ascorbate) is a potent antioxidant that is able to neutralise a wide range of reactive oxygen species, thus protecting host tissues against cell damage and death [5]. Scavenging of reactive oxygen species by ascorbate results in oxidation of the molecule to dehydroascorbic acid (DHA); this is subsequently reduced back to ascorbate by chemical or enzymatic means or, in the absence of sufficient reducing equivalents, DHA breaks down further, resulting in loss of ascorbate from the body [5]. Neutrophils contain high intracellular concentrations of ascorbate and can accumulate more following activation of their oxidative burst, which oxidises extracellular ascorbate to DHA [6]. The DHA is readily accumulated in the cells via glucose transporters and is reduced back to ascorbate intracellularly [6]. Vitamin C has numerous purported roles that support optimal neutrophil function, such as enhancing neutrophil chemotaxis, phagocytosis, the oxidative burst and bacterial killing [7]. Vitamin C may also attenuate necrotic cell death, including NETosis, and support apoptosis and subsequent macrophage clearance of the spent neutrophils, thus facilitating resolution of the inflammatory process [7].

A limited number of studies have assessed the ascorbate status of leukocytes during infections. An early study carried out with leukocytes isolated from people with the common cold showed intracellular ascorbate concentrations decreased by up to 50% during the early stages of the cold [8]. In contrast, another study carried out in children with various infections showed no difference in leukocyte ascorbate concentrations between controls and patients, and no difference during and after the infection, despite plasma ascorbate concentrations being significantly depleted during the infections [9]. Thus, the literature is not clear on the effects of infection on leukocyte ascorbate concentrations. Therefore, we carried out a study with neutrophils isolated from critically ill patients with sepsis to determine their ascorbate status relative to those isolated from healthy controls. We also assessed ex vivo ascorbate uptake by neutrophils from septic patients, since this is negligible in neutrophils from healthy volunteers [10].

## 2. Materials and Methods

Ethical approval was obtained from the New Zealand Health and Disability Ethics Committees for the collection of blood samples from healthy volunteers (URA06/12/083) and from two cohorts of critically ill septic patients in Christchurch Hospital intensive care unit (16NTA238 and 15STH36). Patients were included in the septic cohorts if they met the following criteria: receiving intravenous antimicrobial therapy specifically for infection, receiving ≥ 5 µg/min (≥0.06 µg/kg/min) noradrenaline or adrenaline, evidence of organ dysfunction, i.e., Sequential Organ Failure Assessment (SOFA) score ≥ 2 for at least one of the following: respiratory function (ratio of partial pressure of arterial oxygen and fraction of inspired oxygen [PaO_2_/FiO_2_] < 300), liver function (bilirubin > 33 μmol/L), coagulation (platelets < 100 × 10^3^/μL) or renal function (creatinine > 171 μmol/L).

### 2.1. Blood Cell Differentials and Morphology

Blood samples were collected from the peripheral veins of healthy volunteers (total of *n* = 20) and via an arterial line from septic patients (total of *n* = 18). Patient blood samples were analysed by Canterbury Health Laboratories, an International Accreditation New Zealand (IANZ) laboratory, using flow cytometry to determine cell counts and differentials, and by microscopy following methanol fixation and Wright’s staining to examine cell morphology.

### 2.2. Plasma Ascorbate and Myeloperoxidase Analysis

Non-fasting blood samples (4 mL into lithium heparin) were immediately placed on ice and processed within two hours of collection by centrifuging at 4 °C to separate plasma. Some of the plasma was immediately frozen at −80 °C and the remainder treated with an equal volume of perchloric acid (0.54 M) containing the metal chelator diethylenetriaminepentaacetic acid (DTPA, 100 µmol/L) to precipitate proteins and stabilize ascorbate [11]. The acidified supernatants were stored at −80 °C prior to analysis of ascorbate concentrations by reverse-phase HPLC with electrochemical detection [11]. Plasma myeloperoxidase concentrations were determined using a commercial sandwich ELISA kit (ab119605, Abcam, Melbourne, Australia).

### 2.3. Erythrocyte Isolation and Ascorbate Analysis

Erythrocyte extracts were prepared as described previously [12]. Briefly, following removal of plasma and the leukocyte layer, the erythrocytes were washed with a 10-fold excess of ice-cold PBS containing DTPA (100 µmol/L). The packed cells were then lysed with a four-fold excess of ice-cold water containing DTPA. The lysate was transferred to a centrifugal filter (Amicon Ultra 0.5 mL, 10K Ultracel^®^; Merck Millipore, Burlington, MA, USA) and spun for 20 min at 14,000× *g* at 4 °C to remove the haemoglobin. An equal volume of perchloric acid/DTPA solution was added to the ultrafiltrate and centrifuged, and the supernatant was frozen at −80 °C. The ascorbate content of the erythrocytes was determined using HPLC as described for plasma and corrected for the packed cell content of the lysate by determination of the haemoglobin concentration [13]. Erythrocytes from patients with sepsis can exhibit morphological and other abnormalities [14], and vitamin C deficiency can induce erythrocyte fragility [15]. However, total erythrocytes were collected with this method (no density centrifugation steps were used) and the ascorbate content was also corrected for haemoglobin concentration.

### 2.4. Neutrophil Isolation and Morphology Microscopy

Neutrophils were isolated from the peripheral blood of healthy volunteers using Ficoll-Hypaque and dextran sedimentation, followed by hypotonic erythrocyte lysis, as described previously [16]. Neutrophils were isolated from the blood of septic patients by a modified version of this method. Briefly, after removal of the plasma for ascorbate and myeloperoxidase analyses described above, the remaining blood cells were diluted with phosphate-buffered saline (PBS) to a total of 8 mL and separated by dextran sedimentation (1% dextran) for up 40 min. The upper layer was then removed to a fresh tube and made up to 50 mL with PBS before centrifugation at 1000× *g* for 5 min. The cell pellets were resuspended in PBS and two-fold excess of water was added with gentle inverting for two minutes to lyse the remaining erythrocytes. Sodium chloride (0.25 fold of 2.7%) was added to restore osmolarity prior to centrifugation. The cells were resuspended in Hanks Buffered Saline Solution (HBSS) and were counted using a haemocytometer. Samples from the healthy volunteers were >95% neutrophils and those from the septic patients comprised >92% neutrophils, as determined by flow cytometry.

Analysis of neutrophil morphology was carried out by microscopy. Cells in HBSS were spun onto glass microscope slides (1000× *g* for 5 min) using a Cytospin™ centrifuge (Fisher Scientific, Auckland, New Zealand) then fixed with ice-cold methanol and stained with haematoxylin and eosin. Images were captured with an Olympus CKX53 microscope (Olympus New Zealand, Auckland, New Zealand), using the 40× objective and cellSense Entry Imaging software (Olympus New Zealand, Auckland, New Zealand).

### 2.5. Ex Vivo Ascorbate Uptake by Neutrophils

For ascorbate uptake experiments, the cells were dispensed into sterile 1.7 mL tubes at 5 × 10^6^/mL with either ascorbate or DHA (at concentrations of 50–200 µmol/L, in 1 mL total volume) for the indicated times (15–60 min). Tubes were rotated gently end-over-end at 37 °C. After incubation, cells were centrifuged at 1000× *g* for 5 min and washed with PBS to remove any extracellular ascorbate or DHA and then resuspended in a small volume of PBS. For HPLC analysis of the ascorbate content of the cells, an equal volume of perchloric acid/DTPA solution was added and the supernatant stored at −80 °C prior to analysis by HPLC as described above for plasma.

### 2.6. Ex Vivo Stimulation of Neutrophil Oxidative Burst

As described previously [17], the rate of superoxide generation by activated neutrophils was measured indirectly as a function of cytochrome *C* reduction. Neutrophils (0.5 × 10^6^) were stimulated with phorbol myristate acetate (PMA; 100 ng/mL) in the presence of catalase (20 μg/mL) and cytochrome *C* (40 μmol/L). Activity (μmol superoxide min^−1^/10^6^ cells) was calculated from the change in absorbance at 550 nm (over 5 min at 37 °C) using the extinction coefficient, 21.1 × 10^3^ M^−1^ cm^−1^.

### 2.7. Statistical Analysis

Data are presented as median and interquartile range (Q1, Q3) or mean and standard error of the mean (SEM) as indicated. Differences between groups were determined by unpaired Mann–Whitney U, paired t-tests or paired Wilcoxon signed-rank tests, as indicated, with *p* < 0.05 signifying statistical significance. Statistical analyses were carried out using GraphPad Prism 9 software (GraphPad, San Diego, CA, USA).

## 3. Results

### 3.1. Characteristics of the Septic Participants

The characteristics of the cohort of septic participants (*n* = 18) are shown in Table 1. The participants were predominantly male (78%) and elderly (median age of 66 years). The primary source of sepsis was abdominal (50%), followed by pulmonary (28%). Most of the participants (89%) required mechanical ventilation.

### 3.2. Neutrophil Ascorbate Content Relative to Plasma Concentrations

**Figure 1:**
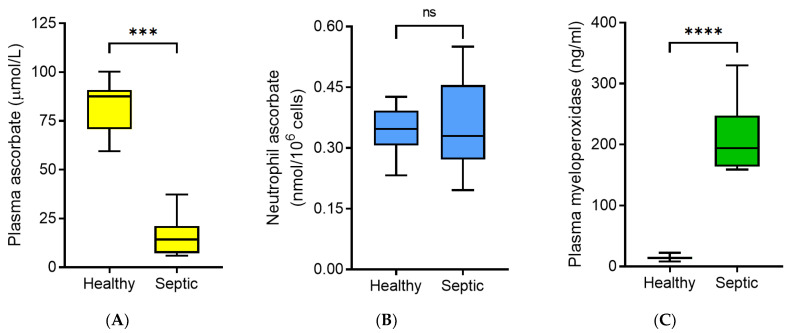
Ascorbate and myeloperoxidase concentrations in healthy volunteers and septic patients. The ascorbate concentrations of (**A**) plasma and (**B**) neutrophils from healthy volunteers (*n* = 7 for plasma and *n* = 13 for neutrophils) and septic patients (*n* = 9 paired plasma and neutrophil samples) were determined by HPLC. (**C**) Plasma myeloperoxidase concentrations from healthy volunteers (*n* = 13) and septic patients (*n* = 9) were determined by ELISA. Box plots show medians with 25th and 75th percentiles as boundaries and whiskers indicate the range. Mann–Whitney U tests indicated significant differences for plasma ascorbate (*** *p* < 0.001) and myeloperoxidase (**** *p* < 0.0001), but not significant (ns) for neutrophil ascorbate (*p* > 0.05).

The median non-fasting plasma ascorbate concentration of the septic patients was 14 (7.2, 21) µmol/L, which was significantly lower than that of healthy volunteers (88 (71, 91) µmol/L, *p* < 0.001; Figure 1A). Unexpectedly, the median intracellular ascorbate concentration of neutrophils isolated from the septic patients (0.33 (0.27, 0.46) nmol/10^6^ cells) was comparable to that of healthy volunteers (0.35 (0.31, 0.39) nmol/10^6^ cells, *p* > 0.05; Figure 1B). This was despite the patients as a group presenting with hypovitaminosis C (plasma concentration < 23 µmol/L). The neutrophil enzyme myeloperoxidase is an in vivo source of oxidants, such as hypochlorous acid, which can readily oxidise ascorbate to DHA [18]. We observed significantly higher concentrations of myeloperoxidase in the circulation of the septic patients (194 (164, 248) ng/mL; *n* = 9) relative to healthy controls (14 (12, 16) ng/mL; *n* = 13; *p* < 0.0001; Figure 1C).

### 3.3. Erythrocyte Ascorbate Content Relative to Plasma Concentrations

**Figure 2:**
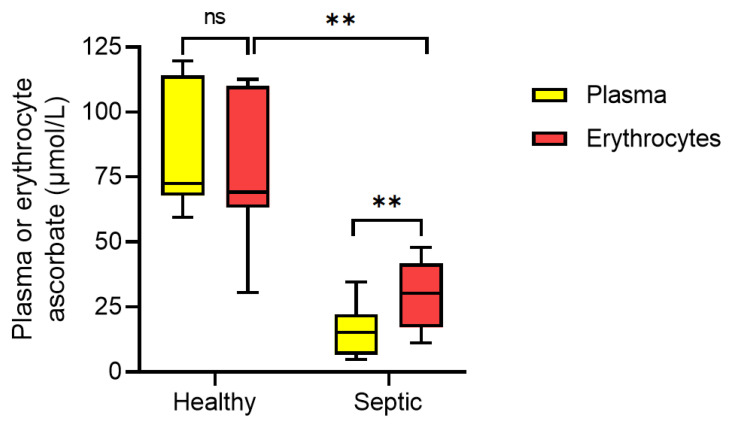
Erythrocyte ascorbate content relative to plasma concentrations. The ascorbate status of plasma (yellow bars) and erythrocytes (red bars) from healthy volunteers (*n* = 7) and septic patients (*n* = 9) was determined by HPLC. Box plots show medians with 25th and 75th percentiles as boundaries and whiskers indicate the range. ** *p* = 0.002 for healthy vs. septic erythrocyte ascorbate concentrations (Mann–Whitney U test) and *p* = 0.008 for paired septic plasma and erythrocyte ascorbate concentrations (Wilcoxon paired signed-rank test).

Erythrocytes lack vitamin C transporters (SVCT) and exclusively accumulate ascorbate in the form of DHA via glucose transporters [19]. In contrast to neutrophils, the median ascorbate concentration of erythrocytes from septic patients (30 (17, 42) µmol/L) was significantly lower relative to those from healthy volunteers (69 (65, 110) µmol/L, *p* = 0.002; Figure 2). However, there was a 2.2-fold higher concentration of erythrocyte ascorbate relative to matched plasma samples in the septic patients (*p* = 0.008). Microscopy indicated some evidence of erythrocytes with morphological abnormalities, including anisocytosis, elliptocytes, acanthocytes (spur cells) and echinocytes (burr cells) in the blood from 7 of the 9 septic patients.

### 3.4. Ex Vivo Ascorbate Uptake by Neutrophils from Septic Patients

**Figure 3:**
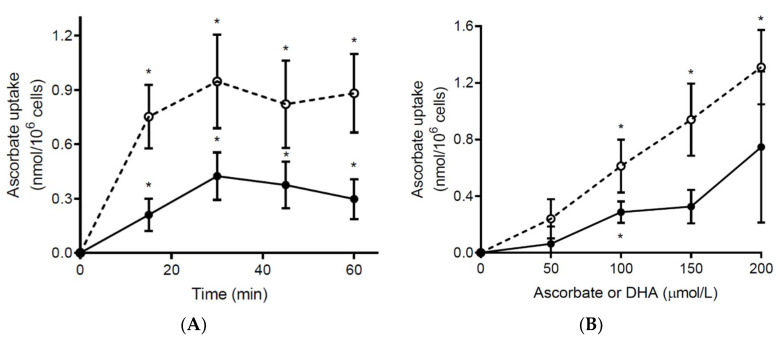
Ascorbate uptake by neutrophils from septic patients. The patients’ neutrophils (5 × 10^6^/mL) were incubated with ascorbate (● solid line) and DHA (◯ dashed line) at (**A**) a concentration of 200 µmol/L for the indicated times (*n* = 4; baseline ascorbate = 0.43 nmol/10^6^ cells), or (**B**) at the indicated concentrations for 30 min (*n* = 3; baseline ascorbate = 0.46 nmol/10^6^ cells). Intracellular ascorbate uptake was determined using HPLC. Data represent mean and SEM, * indicates a significant difference from baseline at *p* < 0.05 (paired *t*-test).

We have shown previously that neutrophils isolated from healthy volunteers uptake negligible extracellular ascorbate unless their oxidative burst is stimulated; this oxidises the ascorbate to DHA, which is rapidly accumulated by the cells via glucose transporters [10]. In contrast to neutrophils from healthy volunteers, we found that unstimulated neutrophils from septic patients were able to accumulate ascorbate in a time and concentration-dependent manner, despite containing comparable baseline ascorbate concentrations to neutrophils from healthy individuals (Figure 3). Incubation with an equivalent concentration of DHA is shown for comparison; although the ascorbate content of the cells approximately doubled, this was still only 35 (24, 41)% of the total DHA uptake observed.

### 3.5. Morphology and Ex Vivo Activation of Neutrophils from Septic Patients

**Figure 4:**
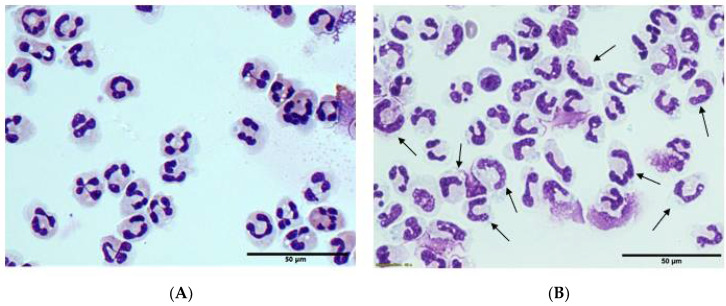
Morphology of neutrophils isolated from (**A**) healthy volunteers and (**B**) septic patients. Arrows indicate examples of immature ‘band cells’ that lack segmented, multi-lobed nuclei. Representative images captured using the 40× objective (scale bar = 50 µm). Cells were stained with hematoxylin and eosin.

Neutrophil counts were dramatically elevated in all of the septic patients: 15 (11, 24) × 10^9^/L (normal range: 1.9–7.5 × 10^9^/L). Flow cytometry showed a ‘left shift’ of the neutrophils from the septic patients indicating a higher proportion of immature cells. Microscopy confirmed a high proportion of neutrophils with immature ‘band’ morphology (Figure 4). There was also evidence of toxic granulation in 4 of the 9 patient samples. Metamyelocytes (immature granulocytes) were also elevated in the septic patients: 0.2 (0.1, 0.8) × 10^9^/L (normal range: 0–0.06 × 10^9^/L). Neutrophils from septic patients can exhibit enhanced basal oxidant production, but diminished ability to generate oxidants upon ex vivo stimulation of their oxidative burst [20]. We observed that neutrophils from septic patients tended to have a diminished ability to generate oxidants following stimulation with a phorbol ester relative to those from healthy volunteers (4.5 (1.8, 6.6) vs. 8.4 (5.6, 8.6) nmoles superoxide/min/10^6^ cells).

## 4. Discussion

To our knowledge, this is the first study to assess the ascorbate content of neutrophils from critically ill patients with septic shock. Despite median plasma ascorbate concentrations being in the hypovitaminosis C range, the neutrophils isolated from the septic patients had comparable intracellular ascorbate concentrations to those from healthy volunteers. The higher than expected ascorbate content of the neutrophils isolated from the septic patients could indicate enhanced systemic oxidative stress that would potentially result in elevated concentrations of DHA, which is readily accumulated and reduced back to ascorbate by neutrophils [6]. The septic patients had elevated neutrophil counts and we also demonstrated significantly elevated circulating concentrations of the neutrophil enzyme myeloperoxidase, which generates the potent oxidant hypochlorous acid that can readily oxidise ascorbate [18]. Furthermore, we have previously demonstrated elevated markers of protein oxidation in the circulation of patients with pneumonia and sepsis [3,21], confirming enhanced oxidative stress in patients with severe infections.

Although an early study reported elevated DHA concentrations in the plasma of patients with pneumonia and other infectious diseases [22], this was likely an ex vivo artefact [23]. We and others have not previously detected significant concentrations of DHA in the plasma of septic patients [4,24]. This can be explained by the rapid uptake of DHA by leukocytes and other cells of the vasculature via their glucose transporters [6]. In support of this premise, erythrocytes from the septic patients were found to contain 2.2-fold higher concentrations of ascorbate than those in matched plasma. Since erythrocytes lack SVCT and exclusively accumulate ascorbate in the form of DHA, our findings suggest that there has been some generation of DHA in the plasma of the critically ill patients.

The formation of DHA in patients with severe infections could explain some of the discrepancy in results of the earlier leukocyte studies, one of which showed decreased leukocyte ascorbate content during the common cold [8], and the other which showed no difference in leukocyte ascorbate content in other more severe infections [9]. The latter study showed significantly depleted plasma vitamin C concentrations in most of the severe infections relative to healthy controls, supporting the involvement of oxidative stress and likely import of DHA into leukocytes. Like the earlier Hume and Weyers study [8], they also reported depleted leukocyte ascorbate concentrations, but not plasma ascorbate concentrations, in a subgroup of cases with the common cold [9]. This suggests that the common cold may not be severe enough to stimulate significant systemic oxidative stress.

Interestingly, despite comparable intracellular ascorbate concentrations to healthy controls, ex vivo incubation of the neutrophils from septic patients with added ascorbate provided further increases in intracellular ascorbate concentrations. Previously, we have shown minimal uptake of ascorbate by neutrophils from healthy volunteers unless their oxidative burst was stimulated [10]. Neutrophils from septic patients exhibit elevated basal oxidant production, but attenuated ex vivo activation of their oxidative burst [20]. This could be due to the higher proportion of immature ‘band cells’ present in these samples, as these cells have been demonstrated to have less efficient phagocytosis and bacterial killing via reactive oxygen species [25]. Alternatively, the neutrophils isolated from septic patients may already be in an activated state. Enhanced in vivo activation of neutrophils in septic patients is supported by the significantly elevated plasma concentrations of myeloperoxidase relative to healthy controls observed in this study and previous research [26].

Neutrophils from septic patients may also have upregulation of the vitamin C transporter SVCT2. Although haematopoietic stem cells and haematopoietic progenitor cells have significantly elevated expression of SVCT2 and high intracellular concentrations of ascorbate, this is diminished following differentiation to the common myeloid progenitor and granulocyte–macrophage progenitor [27], and is therefore unlikely to be a characteristic of the immature cell types observed in the blood of septic patients. An alternative explanation may be upregulation of SVCT2 transcription following exposure to oxidative stress [28]. Thus, in environments of elevated oxidative stress and concomitant depletion of ascorbate, such as is observed in septic patients, upregulation of leukocyte SVCT2 may be a consequence of, or compensation for, low ascorbate availability.

This premise is supported by a study of elderly patients with severe respiratory infections (bronchitis and pneumonia), which showed comparable mean neutrophil ascorbate concentrations to those observed in our study (0.3 nmol/10^6^ cells) [29]. In line with our ex vivo ascorbate uptake experiments, the ascorbate content of the neutrophils more than doubled following supplementation of the patients with 200 mg/day of vitamin C [29]. This lends support to the premise that patients with severe infections have increased generation of DHA (via neutrophil dependent or independent pathways), or upregulation of neutrophil SVCT2. The latter remains to be confirmed as other research has indicated that inflammatory mediators, which are significantly elevated in septic patients, may inhibit the upregulation of SVCT [30].

Recently, there has been growing interest in the effects of vitamin C administration on patients with severe infections, including coronavirus disease (COVID-19), and sepsis [31,32]. Studies have indicated that neutrophils from patients with severe infections and sepsis have abnormalities of their chemotactic and other antimicrobial functions [33]. Supplementation with vitamin C has been shown to restore these essential neutrophil functions and also resulted in the resolution of recurrent infection in a number of small studies [7]. An RCT carried out in 20 septic patients, half of whom were administered vitamin C at a dose of 450 mg/d, showed effects of vitamin C on various neutrophil apoptotic mediators [34]. None of these studies, however, reported neutrophil ascorbate status before or after supplementation.

## 5. Conclusions

The findings from our study indicate that neutrophils from septic patients exhibit comparable intracellular ascorbate content to those from healthy controls, despite the patients presenting with hypovitaminosis C. The cells also showed elevated uptake of exogenous ascorbate relative to those from healthy volunteers. The mechanisms involved are currently uncertain, but could involve increased generation of DHA in septic patients, enhanced basal activation of their neutrophils, resulting in localised generation and uptake of DHA, or upregulation of their vitamin C transporters, resulting in enhanced uptake of ascorbate. Future trials are needed to ascertain the effect of vitamin C administration on neutrophil ascorbate status and functions in patients with severe infection and sepsis to help elucidate the mechanisms involved.

## Figures and Tables

**Table 1:** Characteristics of the septic patients.

Parameter	Septic Patients (*n* = 18)
Male sex, *n* (%)	14 (78) ^1^
Age, years	66 (62, 77)
Source of sepsis, *n* (%):	
Abdominal	9 (50)
Pulmonary	5 (28)
Skin	2 (11)
Blood	2 (11)
SAPS ^2^ II	50 (44, 56)
APACHE ^3^ III	79 (70, 98)
SOFA ^4^ score	9 (8, 11)
Mechanical ventilation, *n* (%)	16 (89)

^1^ Data represent *n* (%) or median (Q1, Q3). ^2^ SAPS Simplified acute physiology score. ^3^ APACHE Acute physiology and chronic health evaluation. ^4^ SOFA Sequential organ failure assessment.

## Data Availability

The data is contained within the article.
